# Lizard colour plasticity tracks background seasonal changes

**DOI:** 10.1242/bio.052415

**Published:** 2020-06-04

**Authors:** Daniele Pellitteri-Rosa, Andrea Gazzola, Simone Todisco, Fabio Mastropasqua, Cristiano Liuzzi

**Affiliations:** 1 Laboratorio di Zoologia, Dipartimento di Scienze della Terra e dell'Ambiente, Università di Pavia, Pavia 27100, Italy; 2 Societas Herpetologica Italica, Sezione Puglia, Bitritto, BA 70020, Italy

**Keywords:** Colour variation, Crypsis, Dorsal pigmentation, Environment, Reptiles, Season

## Abstract

Environmental heterogeneity on a spatial and temporal scale fosters an organism's capacity to plastically alter coloration. Predation risk might favour the evolution of phenotypic plasticity in colour patterns, as individuals who change colour throughout the year may be able to improve their fitness. Here we explored the change in dorsal pigmentation of the Italian wall lizard (*Podarcis siculus campestris*) at three time points (March, July, October) during a period of activity in a Mediterranean natural area in southern Italy. Following a preliminary investigation conducted in 2018, during 2019 we captured 135 lizards and took a picture of their ventral scales to check for possible recapture over the sessions. Lizard dorsal pictures were collected in the field with the support of a reference chart to quantitatively estimate chromatic variables (hue, saturation and value). At the same time, pictures of the environmental background were collected. Our findings suggest that lizards are capable of altering dorsal coloration during seasonal change. They vary from green at the onset of spring, to brownish in the middle of summer and to a greyish colour in October. This modification closely followed environmental background colour variation and enhanced lizard crypsis during each season.

## Abstract (precis)

**Summary:** Our study shows seasonal variation in lizard dorsal colour as a potential adaptive trait in response to environmental background change, and highlights the importance of collecting colour data throughout the year.

## INTRODUCTION

Intraspecific colour variations have historically attracted much interest, especially for their relation with temperature variation, social communication, or predation risk (Endler, 1981; Hoekstra, 2006; Stuart-Fox and Moussalli, 2009). One possible trigger of chromatic variation is the habitat-specific background colour. When the risk of predation is high, body coloration matching the colour of the background is common among prey species and has been reported in many studies (Endler, 1978; Ruxton et al., 2004). A visual resemblance to the background chromatic characteristics (colour, brightness, or pattern) has been proved to be an adaptive trait, which can significantly decrease the risk of detection by predators (Stuart-Fox et al., 2003). Natural populations provide examples of habitat-specific body colours in a great diversity of taxa, including freshwater fish (Whiteley et al., 2009), frogs (Wente and Phillips, 2003), salamanders (Storfer et al., 1999), turtles (McGaugh, 2008), lizards (Rosenblum et al., 2010) and mice (Hoekstra, 2006). Such studies suggest that the adjustment to habitat-specific variation in colour is mostly a result of selective pressure by predators hunting by sight, leading the population towards an adaptive match to the local background. However, body coloration can also affect the fitness of animals through its effects on thermoregulation (Clusella-Trullas et al., 2009) or social communication (Stuart-Fox and Moussalli, 2009).

Animal colour regulation may be fixed (i.e. constitutive) or expressed over different timescales, from seconds to years. In order to achieve an effective background matching, colour change can also follow seasonal changes. This phenomenon has been observed in vertebrates and invertebrates. The tropical butterfly *Bicyclus anynana* shows seasonal variation in wing patterns, following the temperature, which anticipates seasonal change in vegetation (van Bergen and Beldade, 2019); the snowshoe hare (*Lepus americanus*) goes through seasonal coat colour shifts from brown to white. This change has been recorded to be strongly related to survival (Zimova et al., 2016).

Lizard coloration has been explored throughout numerous ecological and evolutionary studies (Olsson et al., 2013; McLean and Stuart-Fox, 2014; Pellitteri-Rosa et al., 2014), however, seasonal colour change has been rarely explored in this group (Cuervo and Belliure, 2013; Cadena et al., 2018). In this study, dorsal colour variation in *Podarcis siculus campestris* was recorded by sampling individuals of the same population in three different months during the yearly period of activity. We also explored the possible differences between sexes in colour variation, and whether it might enhance concealment by tracking environmental modifications that occur during seasonal changes.

## RESULTS

**Fig. 1.**
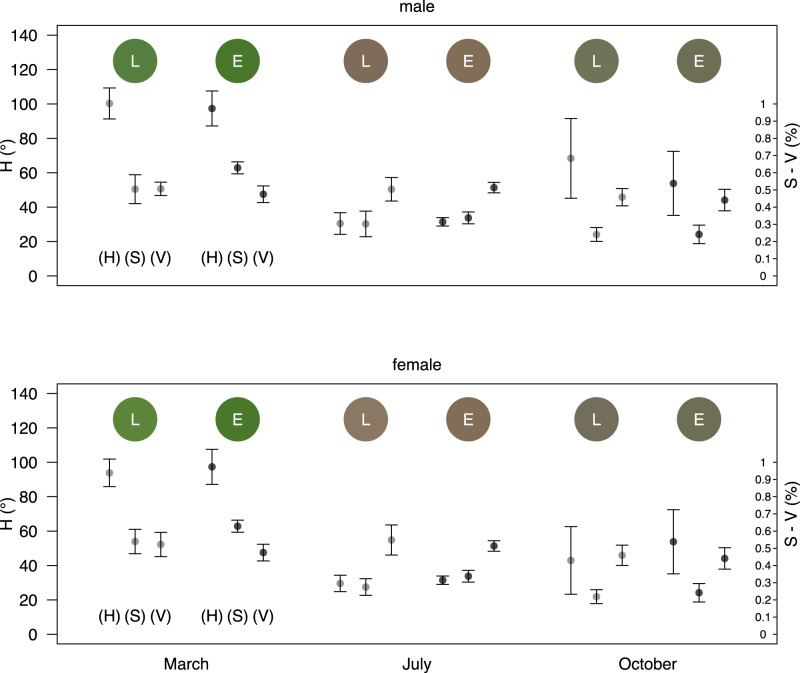
**Means±standard deviations of hue (H), saturation (S) and value (V) for both lizards (light grey) and grassy habitat (dark grey) in 3** **months of data collection in the field (March, *n*=46; July, *n*=48; October, *n*=41) during 2019.** For each plot (males and females), the colours of both lizards (L; left top coloured circular patch) and environment (E; right top coloured circular patch), generated by means values of HSV, are reported. The y-scale on the left is referred to H (°), the one on the right is related to S and V (%).

Hue varied as function of both month (χ^2^=344.87, d.f.=2, *P*<0.001) and in relation to month and sex (month×sex, χ^2^=18.23, d.f.=2, *P*<0.001), showing the highest value during March and decreasing in July and October (Fig. 1), but was not affected by snout-vent length (SVL) (χ^2^=0.11, d.f.=1, *P*=0.73). Male's hue was consistently higher than female's in all months, but with very similar values in July (Fig. 1). Saturation showed a significant effect of month (χ^2^=45.96, d.f.=2, *P*<0.001), but not of sex (χ^2^=1.51, d.f.=1, *P*=0.21) and month×sex interaction (χ^2^=4.20, d.f.=2, *P*=0.12), but the effect of SVL was significant (χ^2^=6.01, d.f.=1, *P*=0.01), and was related to a proportional increase of saturation with SVL (slope±s.e.=0.21±0.09, d.f.=123, t=2.47, *P*=0.015; Fig. 1). Mixed model for value showed a non-significant effect for month, sex and their interaction (χ^2^=1.84, d.f.=2, *P*=0.39; χ^2^=0.01, d.f.=1, *P*=0.92; χ^2^=2.64, d.f.=2, *P*=0.26, respectively); SVL was not significant (χ^2^=2.77, d.f.=1, *P*=0.1) and showed a slight negative relationship with value (slope±s.e.=−0.17±0.10, d.f.=123, t=−1.66, *P*=0.09; Fig. 1).

Background hue and saturation strongly varied with month (Kruskal–Wallis rank sum test: χ^2^=42.92, d.f.=2, *P*<0.001 and χ^2^=48.55, d.f.=2, *P*<0.001; Fig. 1). Comparison between months for both variables confirmed highly significant differences (pairwise comparisons – Benjamini-Hochberg adjustment: all *P*<0.001). Background value was different among months (χ^2^=16.75, d.f.=2, *P*<0.001; Fig. 1), however, pairwise comparisons showed weak differences for March and October (*P*=0.07), but significant differences for the others (March–July: *P*=0.01; July–October: *P*<0.001).

In March, the lizard hue matched the hue of the environment (W=450, *P*=0.89), but saturation and value did not (W=77, *P*<0.001 and W=631.5, *P*=0.01, respectively; Fig. 1). Comparison of March hue with other months showed highly significant differences both for July (W=920, *P*<0.001) and October (W=890, *P*<0.001); the March saturation result was different from both July and October (W=898, *P*<0.001 and W=916, *P*<0.001) and March value was different in comparison to October, but similar when compared to July (W=736.5, *P*<0.001 and W=425, *P*=0.62, respectively).

In July, the lizard hue matched the hue of the environment (W=411.5, *P*=0.35) but was highly different in comparison to March and October (respectively, W=898, *P*<0.001 and W=0, *P*<0.001). The lizard mean saturation estimate was different in comparison to the saturation of the environment for all months (W=244.5, *P*=0.001; Fig. 1). Lizard July value was similar to the value of the background (W=558, *P*-value=0.2961) but was different when compared with other month measurements (March and October, respectively: W=683, *P*=0.006 and W=769, *P*<0.001).

All hue, saturation and value matched the environment in October (lowest *P*=0.53). Comparison with other months showed highly significant differences (*P*<0.001) for all variables, with the sole exception of March value which had a result similar to the October value (W=318, *P*=0.15).

**Fig. 2.**
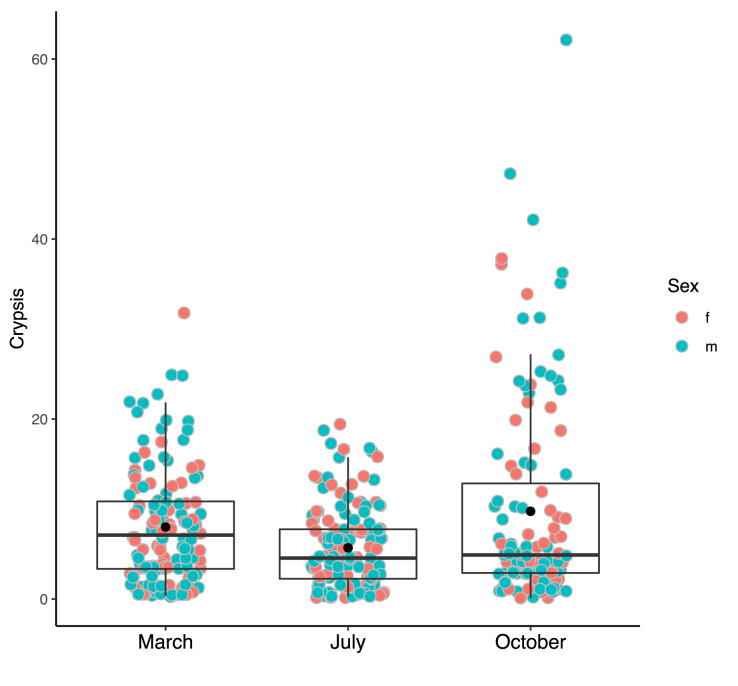
**Degree of crypsis for each month is reported.** Filled black points represent mean crypsis and have been estimated including both sexes.

Crypsis was consistently low in all seasons, with the lowest value occurring in July (means±s.e.=5.69±0.38; Fig. 2). Kruskal–Wallis rank sum test showed significant differences between months (χ^2^=9.02, d.f.=2, *P*=0.01); multiple pairwise comparison (Benjamini-Hochberg adjustment) revealed the only significant difference between March and July (*P*=0.006; Fig. 2).

## DISCUSSION

In this study, we detected a seasonal dorsal colour variation in the Italian wall lizard. Our findings revealed that at the start of spring, animals showed a typical green colouration, corresponding to greater hue values with respect to both those of saturation and value; during summer, lizards exhibited a less lively dorsal colour tending towards a brownish colour, with a strong change of the average hue; finally, at the beginning of autumn, dorsal colouration shifted to greyish colouration with a return to the previous values of hue and a reduction in saturation, which corresponded to a grouping of both green and brownish lizards. Moreover, through image analyses and photographic identification conducted mainly during 2018 and partially in 2019 (few recaptured individuals), we verified that colour varies on an individual basis and, therefore, not only at the population level.

Colour variation is an attracting phenomenon that is widespread in many animal taxa, particularly vertebrates (Galeotti et al., 2003). The adaptive advantages brought by colour change are expected to vary seasonally because of the effect of temperature, variations in reproductive or territorial activity, or predation risk (Cadena et al., 2018). For example, Smith et al. (2016) showed that bearded dragons change colour in order to achieve better thermoregulation, by increasing absorption of solar radiation at low temperatures and decreasing it at high temperatures (see also Clusella Trullas et al., 2007). However, they showed that colours also changed in response to background colour, and this seemed to have a stronger effect than temperature (Smith et al., 2016). In addition, seasonal variation in gonadal hormone levels can strongly affect hormones that stimulate colour change (Nery and de Lauro Castrucci, 1997). Many cases of seasonal chromatic variation linked to the reproductive period are known among lizards, both in males and females (Díaz et al., 1994; Weiss, 2002). However, the colour tends to mainly change in one of the two sexes, with no variation or less intensity in the other one (Cuervo and Belliure, 2013). Moreover, the integument involved in the seasonal colour change is generally that of throat, ventral or flank scales, but in almost no cases of the dorsal surface (Salvador et al., 1997; Galán, 2000).

**Fig. 3.**
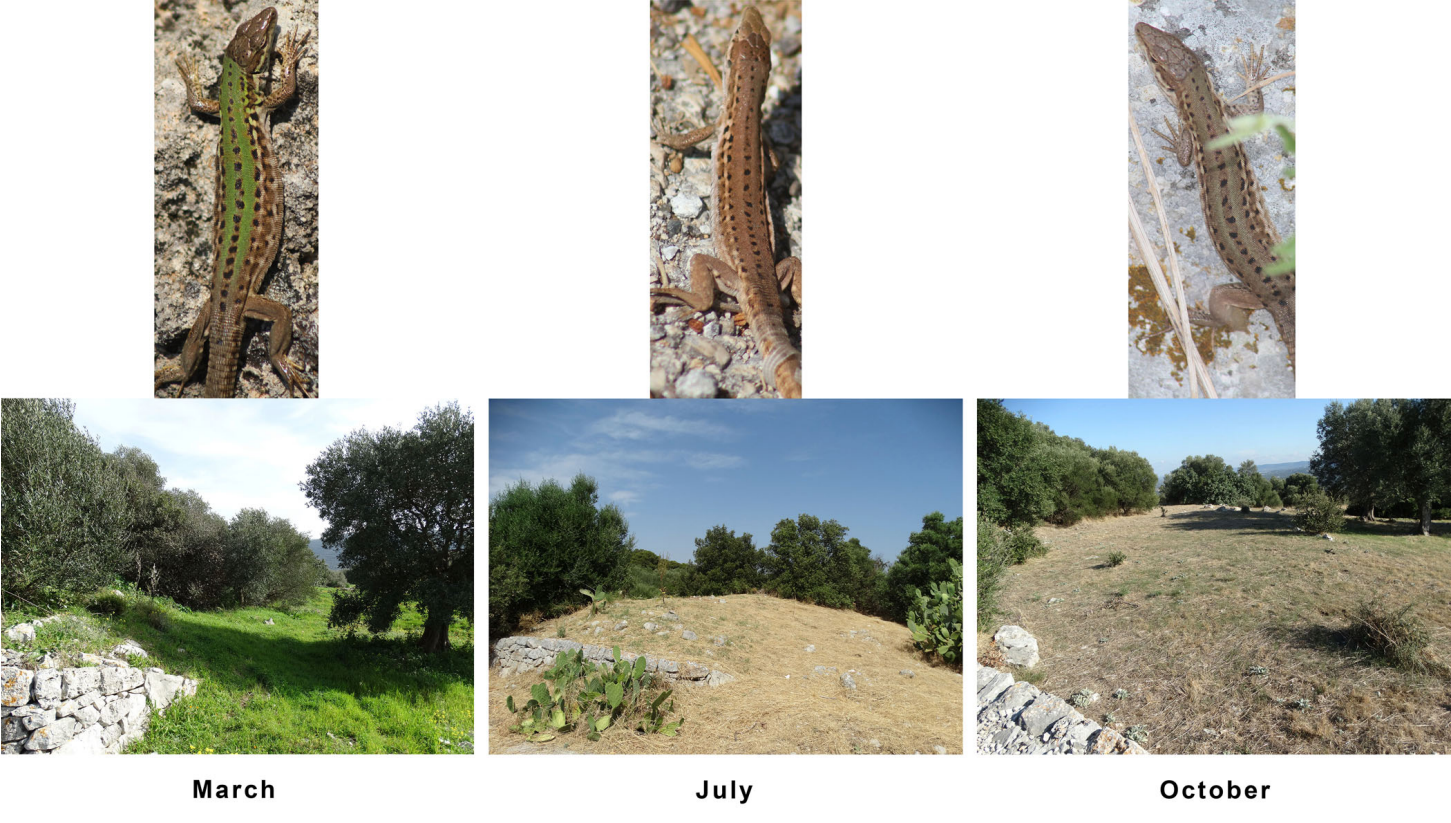
**Matching of colour variation of lizards and grassy habitats throughout the different seasons.** The lizard in the picture is the same female individual photographically recaptured in all the three sampling sessions.

In *P. siculus campestris*, as for other lizards, seasonal changes in dorsal colouration can take up to months to achieve the seasonal chromatic peak, depending on the increase or decrease in pigment concentration (Saenko et al., 2013). In colour-changing ectotherms like this, experiencing high shifts in background colour or potential predators over different seasons, colour variation is mainly considered as a response to background colour than body temperature or social communication (Smith et al., 2016). In our study, the observed sharp change in dorsal colouration throughout the seasons might be associated with an anti-predatory adaptation. This response is performed by displaying a colouration resembling that of those backgrounds where they are the most exposed to potential predators (Merilaita et al., 2017). Interestingly, we found a similar seasonal trend in background mean values of hue, thus, revealing an environmental colour matching with lizard dorsal tonality. The grassy habitat, which in our study area is the main microhabitat used by lizards for thermoregulation, radically changes between spring and summer. The landscape goes from a bright and intense green in spring to a brownish colour in summer due to the dry and arid grass (Fig. 3). In late summer, with the onset of the first rains, the grass begins to regain a greenish colour, creating a mosaic of colours ranging from brown to green, giving an overall shade of grey to the environment. Mean values of chromatic variables for both the lizards and the grassy habitat clearly show that lizard colouration strongly matched seasonal changes of the environment, providing arguments for adaptive cryptic adjustment (West-Eberhard, 2003). The characteristic Mediterranean area considered in this study also included stonewalls, potentially useful for lizard thermoregulation, though less frequently used by them. In contexts where a potential prey moves on different backgrounds, a camouflage strategy can be adopted only on a singular background, for example, the most frequent in the environment or where they are more visible to predators (Michalis et al., 2017; Moreno-Rueda et al., 2019).

We also found interesting results concerning both sex and size/age in relation to colour variation. Although both sexes showed similar trends in chromatic variables throughout the sessions, male hue was significantly higher than female hue in all months, with very similar values in July. In the biophysics of colouration, hue is generally considered as the purest form of a colour having full saturation, described by its dominant wavelength. The other two chromatic variables (i.e. saturation and value) define the brilliance and intensity or refer to the lightness or darkness of a colour, respectively. Therefore, the evidence of a higher hue in males than in females could be associated to a high level of testosterone production in male lizards, as found in birds and reptiles (Stoehr and Hill, 2001; Pérez i de Lanuza et al., 2014). In addition, these differences were higher during reproductive periods (spring and autumn; see Fig. 1), in which males tend to be more competitive for both females and territories access (Marler and Moore, 1988). However, although these differences reflect a certain season-dependent sexual dimorphism in the dorsal colouration, the seasonal colour trend is still the same in both sexes. This suggests that dorsal colour variation may be a generalised phenomenon in this species, without implications related to differential sex-dependent strategies. Moreover, saturation appeared to be affected by size, thus indicating a possible effect of the individual's age on colour expression, as seen in other lizard species (Molnár et al., 2012; Martin et al., 2013).

In conclusion, our study showed a seasonal variation in the dorsal pigmentation of a rather common lizard species. Furthermore, from the parallel analysis of the seasonal variation of the background, we have been able to demonstrate the high chromatic adaptation of lizards to the environment that changes over the seasons. In addition to being a fascinating phenomenon from an evolutionary perspective, to our knowledge it has been rarely observed in reptiles, particularly in lizards, and related solely to different habitat or elevations (Marshall et al., 2015; Moreno-Rueda et al., 2019), but not to season. Our results should be taken into account since many studies have described lizard taxonomic units based on chromatic patterns of a single season (Capolongo, 1984). Systematic studies should probably be reconsidered, in view of possible colour changes throughout different seasons.

## MATERIALS AND METHODS

### Preliminary observations: 2018

During 2018, we conducted preliminary field observations of adult lizards in a population from central Apulia (southern Italy) throughout their period of activity. The sampling site is included in the European special area of conservation (SAC) ‘Murgia dei Trulli’, whose landscape is singularly characterised by typical dry constructions with conical roofs (‘trulli’), surrounded by oak trees, olive groves and a large covering of grassy vegetation. Observations were done by collecting dorsal pictures (Sony HX300 resolution 5184×3888 pixels) without capturing the lizards, from a distance to allow for a good resolution of the collected images (about 1–2 m, using the zoom function, if required). Lizards were photographed over three different seasons (spring, summer and autumn), for a total of 356 collected pictures. Based on a careful visual inspection of photos, in particular the arrangement of the dorsal melanin stains that clearly distinguish each individual, we were able to recognise 26 recaptured individuals (59 out of 356 total pictures). This allowed us to assess a noticeable individual seasonal colour variation, based solely on human visual perception (see some examples of recaptured individuals in the Supplementary Material, S1). However, although this approach has allowed us to notice a clear seasonal colour change of both population and individuals, it has not provided us with quantitative information on their variation in the chromatic characteristics.

### Lizard capture and picture collection: 2019

In order to quantitatively evaluate colour variation, in 2019 we collected adult lizards from the same population during three capture sessions (each session lasted 2–3 days) temporally distributed as follows: March (late winter, early spring), July (full summer) and October (early autumn). The animals were captured by noosing and individually kept in cloth bags until the end of sampling. The lizards were then sexed and measured using a digital calliper (accuracy±0.1 mm) for the SVL. As for many lizard species, males are clearly distinguished from females both in their larger body and head dimensions and in their well-developed femoral pores (Martín et al., 2008). The belly was photographed to check for possible recaptures over the sessions by means of the I^3^S software on the ventral scales (see Sacchi et al., 2010 for more details). This approach is useful to match the images through a geometric method, based on the connection points among lizard scales as descriptors. A matching algorithm using the distribution of these points is highly useful when comparing the images of different individuals. Unlike 2018, we preferred to use this method because it allows the recognition of those individuals with no pattern of melanin spots, known as concolor, which are not detectable by a simple visual method. However, we recaptured only four lizards in two different sessions, but no lizards were recaptured in all three sessions.

**Fig. 4.**
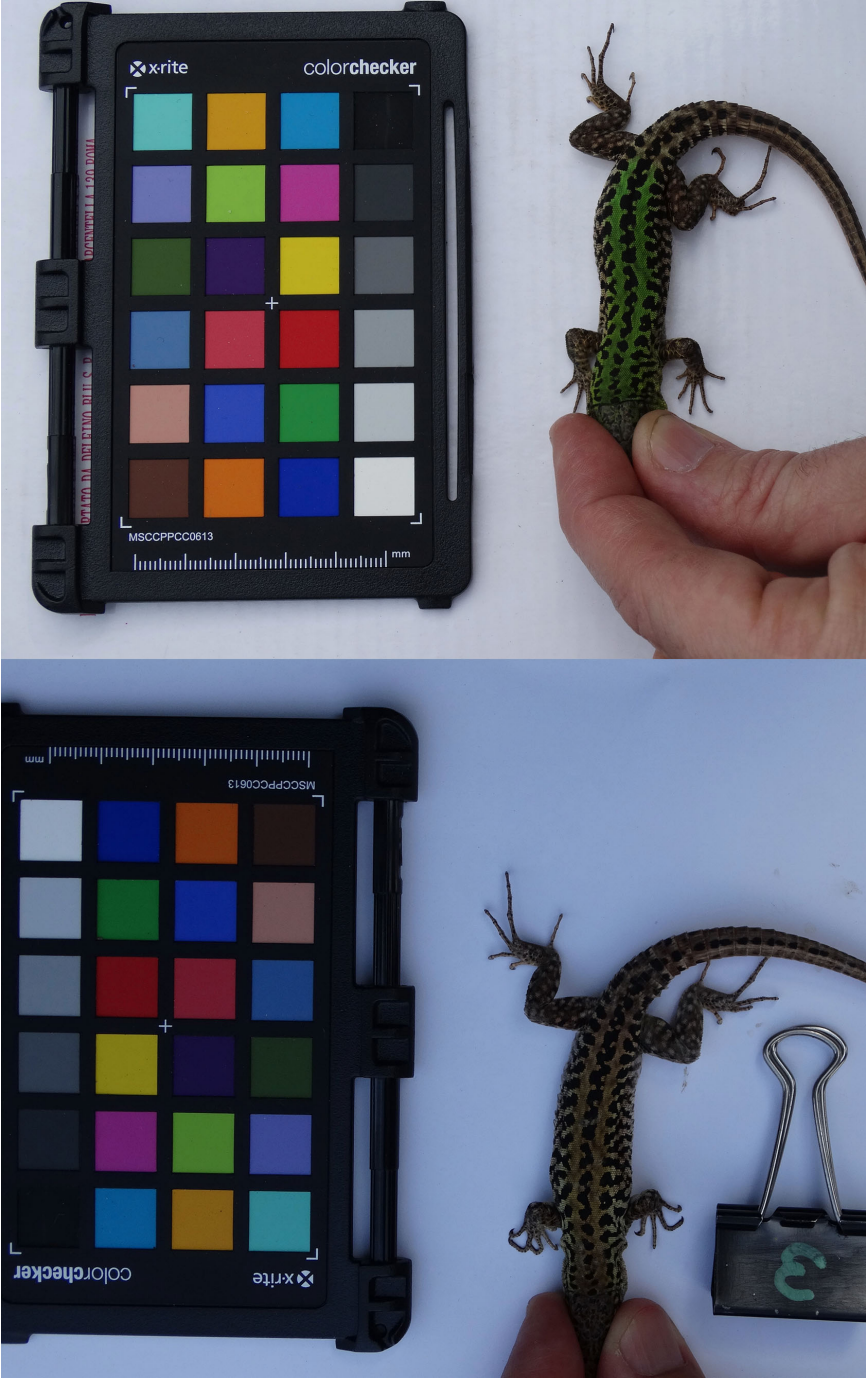
**Male captured in March (top) and recaptured in July 2019 (bottom), photographed adjacent to a GretagMacBeth Mini Color Checker chart.**

Since the human eye perceives colours differently under different light conditions (Endler, 1990), an alternative and very effective system aimed at measuring colour has been validated in recent years (Bergman and Beehner, 2008; Sacchi et al., 2013). Many methods are based on digital photography combined with software programs that allow researchers to objectively quantify colour, being more sensitive than human vision at detecting differences in colour (Villafuerte and Negro, 1998). The method we adopted in this study is quite simple, requiring a digital camera, a colour standard reference (i.e. the Mini Colour Checker chart), and colour analysis software. The most significant advantage of this approach is that it can be applied under natural light conditions and to subjects in their natural habitat, thus standardizing the different light conditions that can be found in wild studies (Bergman and Beehner, 2008). In order to measure the colour variation through the seasons, we took a picture of the dorsal body region of each lizard adjacent to a GretagMacBeth Mini Color Checker chart (24 colour references, 5.7 cm×8.25 cm; Fig. 4). Each lizard was photographed at a distance of about 30 cm.

**Fig. 5.**
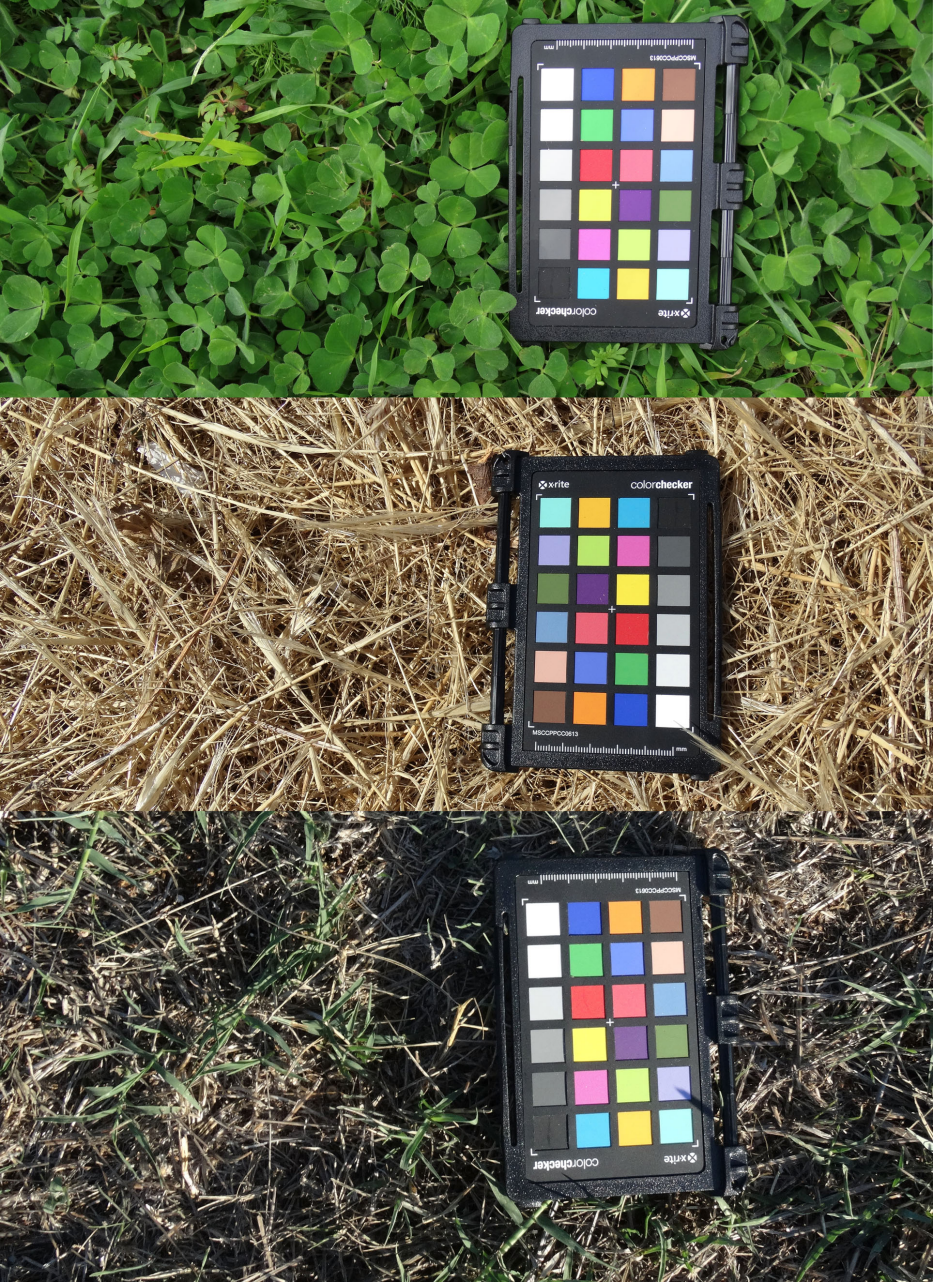
**Grassy vegetation photographed in March (top), July (middle) and October (bottom) 2019.**

During each sampling session, we also took 20 pictures of grassy vegetation adjacent to the Mini Color Checker chart, at a distance of about 50 cm from the photo camera (Fig. 5). We selected the vegetation spots based on the areas where lizards were present, or because they were captured at that point, or because they were in adjacent areas (for example above a stone or a wall). This was carried out to quantitatively characterise the vegetation cover colour representing the entire area considered in the sampling. The lizards were then released at the exact site of capture. Overall, in 2019 we captured 135 lizards (80 males and 55 females, mean SVL±s.e.=74.88±0.54 mm and 63.37±0.65 mm, respectively), well distributed over the seasons (March: 46; July: 48; October: 41).

### Colour measurement

We used RGB values both for lizards and the environment by adopting the method initially proposed in monkeys (Bergman and Beehner, 2008) and later also used in reptiles (Sacchi et al., 2013). The RGB system is an additive colour method in which red (R), green (G) and blue (B) light are mixed together in various ways to reproduce a wide range of colours (for example, a green surface corresponds to the following RGB values: low R and B, high G). It is considered as effective as spectrophotometry in analysing pure colours, but, contrary to spectrophotometry, it allows us to capture the discrete and heterogeneous spatial distribution of an array of different colours, such as those of lizard's dorsal body region, proving to be very efficient in detecting even slight chromatic variations (Villafuerte and Negro, 1998; Sacchi et al., 2013).

We used the Camera plug-in for Adobe Photoshop CS6 to create a new colour profile that adjusted the colour in the photographs (in the tiff format) to the known colour levels in each square of the ColorChecker chart. For each lizard, we measured the colour of the dorsal part by selecting the areas of all scales showing colouration (i.e. black spots were excluded), using the ‘magic wand’ tool (on average roughly 93,000 pixels) and recording the RGB levels using the histogram palette. Through the adoption of a specific conversion algorithm (Chernov et al., 2015), the RGB colour values were finally rearranged in the hue, saturation and value (HSV) system, which is an alternative representation of the RGB colour model. In this system, the colours of each hue are arranged in a radial slice, around a central axis of neutral colours ranging from black at the bottom to white at the top. The HSV representation models the way in which hues of different colours mix together, with the saturation dimension reflecting various brightly coloured hues and the value dimension reflecting the mixture of those tinged with varying amounts of black or white colours. Although the methodology to measure animal colour has been currently moved to use visual models (Delhey et al., 2015; Bitton et al., 2017), the HSV system is still considered one of the most common, intuitive and perceptually relevant representation of points in an RGB colour model. Finally, in our study we did not consider ultraviolet (UV) colourations since they are known to have their maximum reflectance in the flanks (for examples in blue patches), in the head or in ventral scales with respect to dorsal parts (Pérez i de Lanuza, 2019) and are related more to intraspecific communication or thermoregulation (Bajer et al., 2012; Pérez i de Lanuza, 2014).

### Statistical analyses

With the aim to explore the seasonal change in lizard dorsal colour, we adopted linear mixed models that included SVL as covariate, and sex, month and their two-way interaction as fixed factors. Date of collection was included as a random factor. We ran three models with hue, saturation and value as response variables, respectively. In order to meet the assumption of residuals homogeneity, we included a variance structure for a different spread per stratum (i.e. month; Zuur et al., 2007). This resulted in a better fit and lower AIC of the model. In all the models, we assumed a normal error distribution. Models were conducted the package ‘nlme’ (Pinheiro et al., 2019) and type II tests performed with the ‘Anova’ function from the package ‘car’ (Fox and Weisberg, 2019). In order to account for departures from normality, comparisons between lizard and background colour for each month were obtained through a series of Wilcoxon rank sum tests for hue, saturation and value, respectively (sex was excluded from the analysis). Kruskal–Wallis rank sum test was used to explore differences of hue, saturation and value of the background and crypsis differences among months; pairwise comparisons were eventually calculated by using Wilcoxon rank sum test with Benjamini-Hochberg adjustment.

When no information on the visual system of potential predators is available, the difference between animal and background colours can provide some information on prey degree of crypsis. Colour dissimilarity can be measured as the Euclidean distance between colours components with a simple formula: 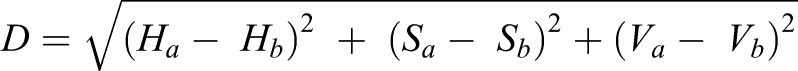 (Endler, 1990; Moreno-Rueda et al., 2019). Subscripts a and b refer to animal and background, respectively. In our study, the mean background colour components were calculated from a sample of 20 pictures collected in each different month (March, July, October), and in the same location. Each component of the distance (ΔΗ, ΔS, ΔV) was so obtained as to correspond to the difference between each lizard's specimen dorsal colour and the mean calculated for the colour of the background in a given month (see also Moreno-Rueda et al., 2019). In the formula, we inserted the mean value of each background component and the value of each lizard dorsal component, and distance estimates were calculated for each month. A low D value reveals a good animal-background match, a high one a weak match.

Statistical analyses were performed using R Version 3.6.0 (R Core Team, 2019).

### Ethics

All lizards captured in this study were kept in cloth bags during 1-day sessions and later released at the exact site of capture, thus minimizing the disturbance to their biorhythms. This study was realised in conformity with the current Italian laws (DPN/II DIV/45377/PNM/2019).

## Supplementary Material

Supplementary information
