# Supplementary material for: Lizard colour plasticity tracks background seasonal changes
Source: Biol Open. 2020 Jun 4;9(6):bio052415. doi: 10.1242/bio.052415 (PMC7286296; doi:10.1242/bio.052415)
Supplement: Supplementary information [file biolopen-9-052415-s1.pdf]

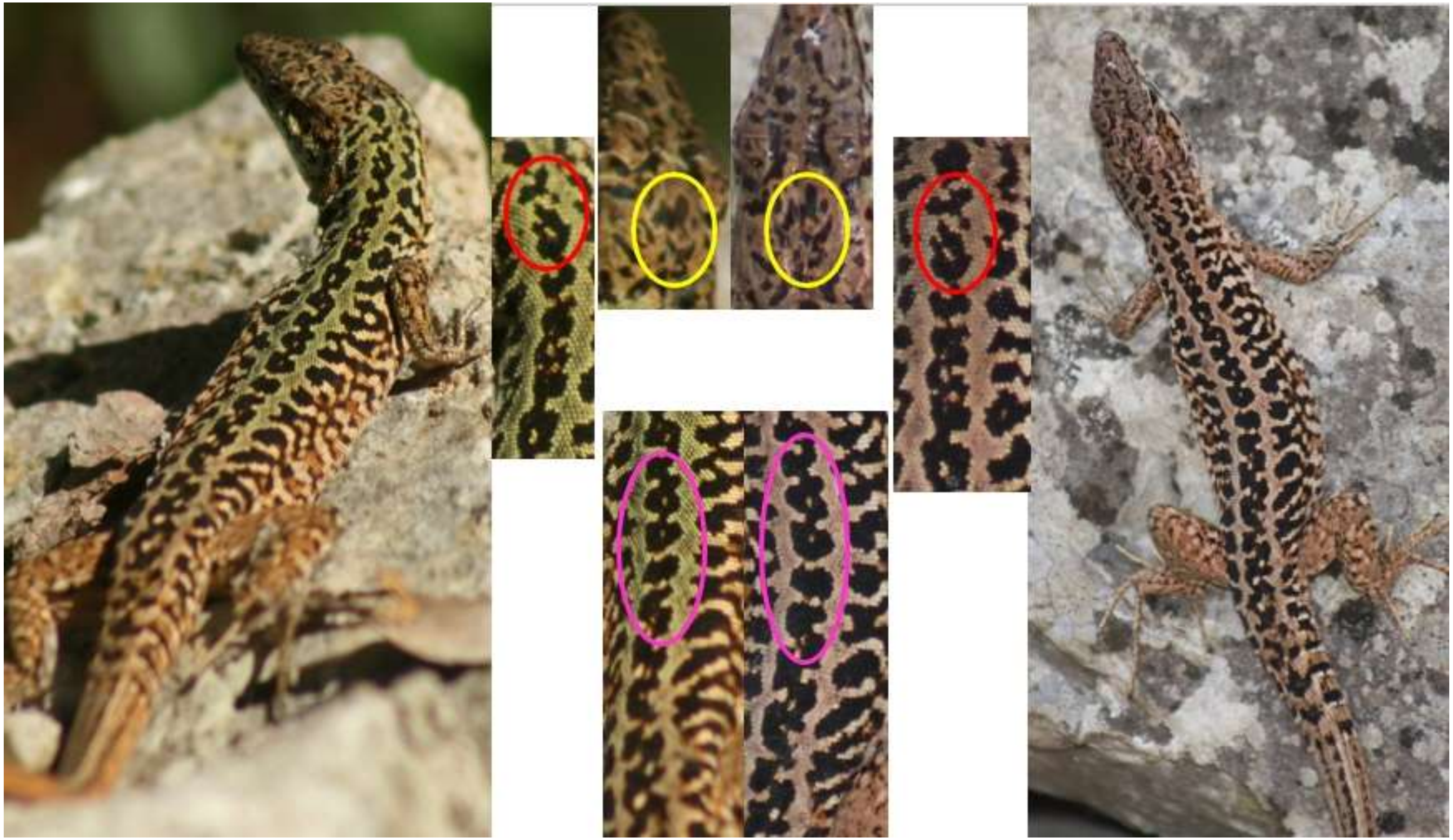

Figure s1.2 Example of a male photographically captured in spring (left) and recaptured in summer (right), recognized through an accurate visual inspection based on the arrangement of the dorsal melanin stains that clearly distinguish each individual.

Biology Open • Supplementary information

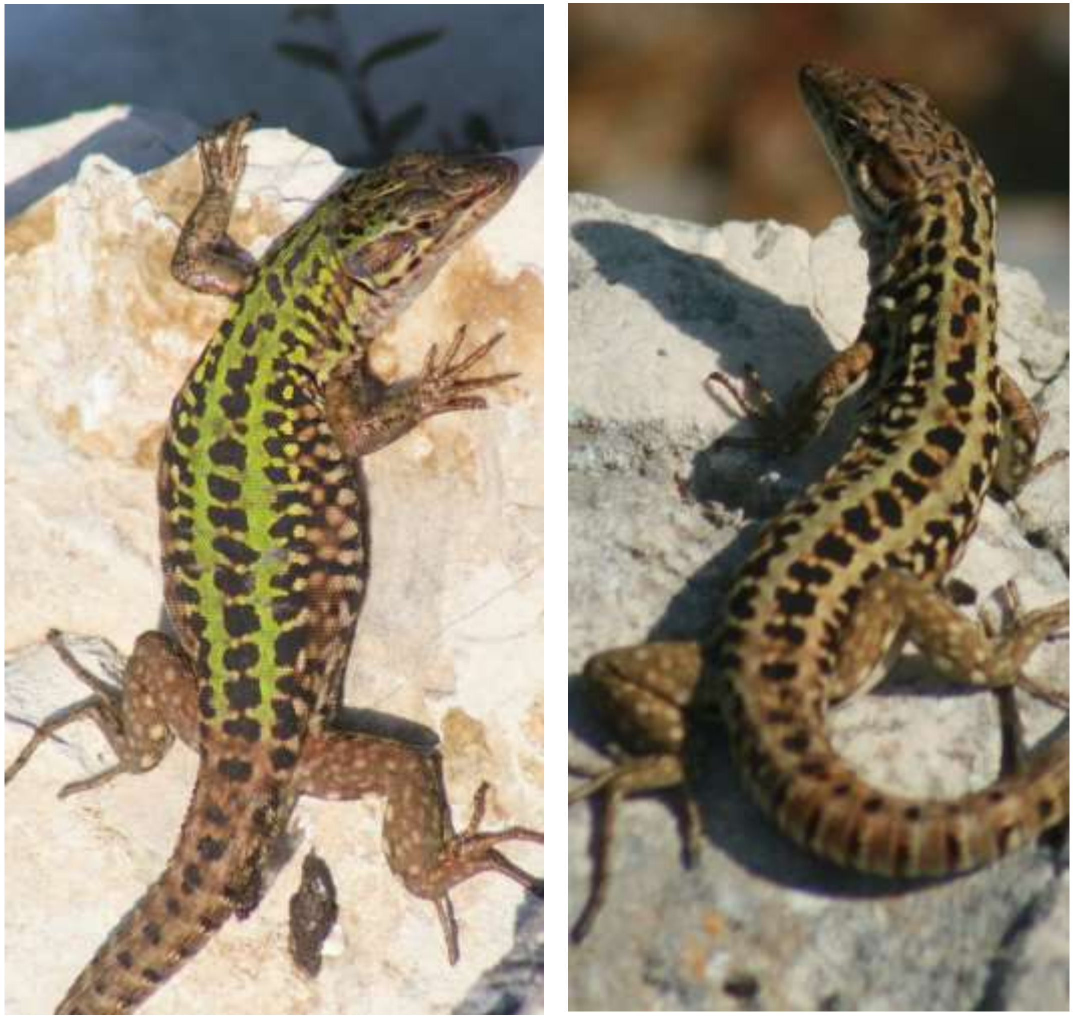

Figure s1.2 Male photographically captured in March (left) and recaptured in June 2018 (right).

Biology Open • Supplementary information

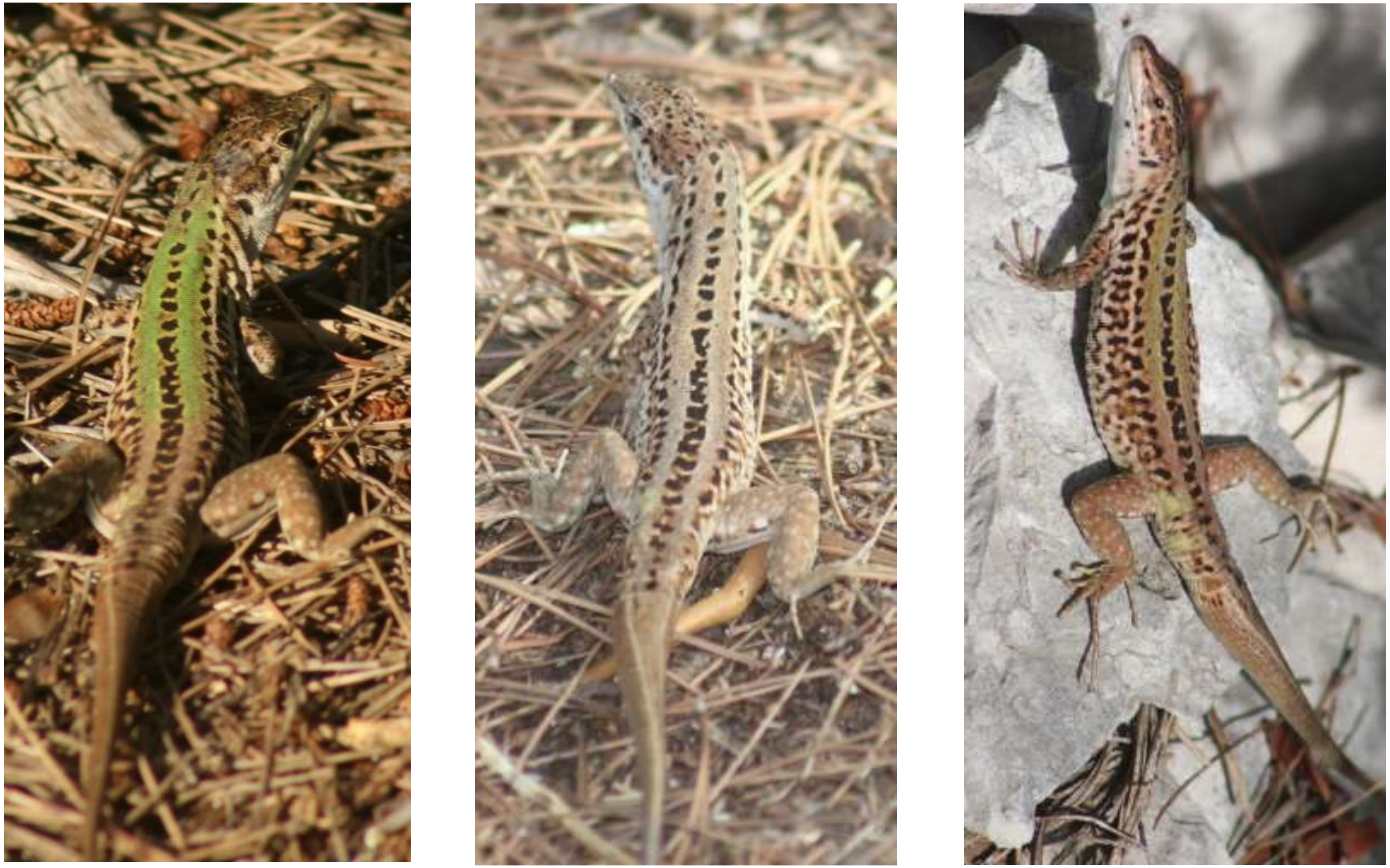

Figure s1.3 Male photographically captured in March (left) and recaptured in August (middle) and October 2018 (right).

Biology Open • Supplementary information

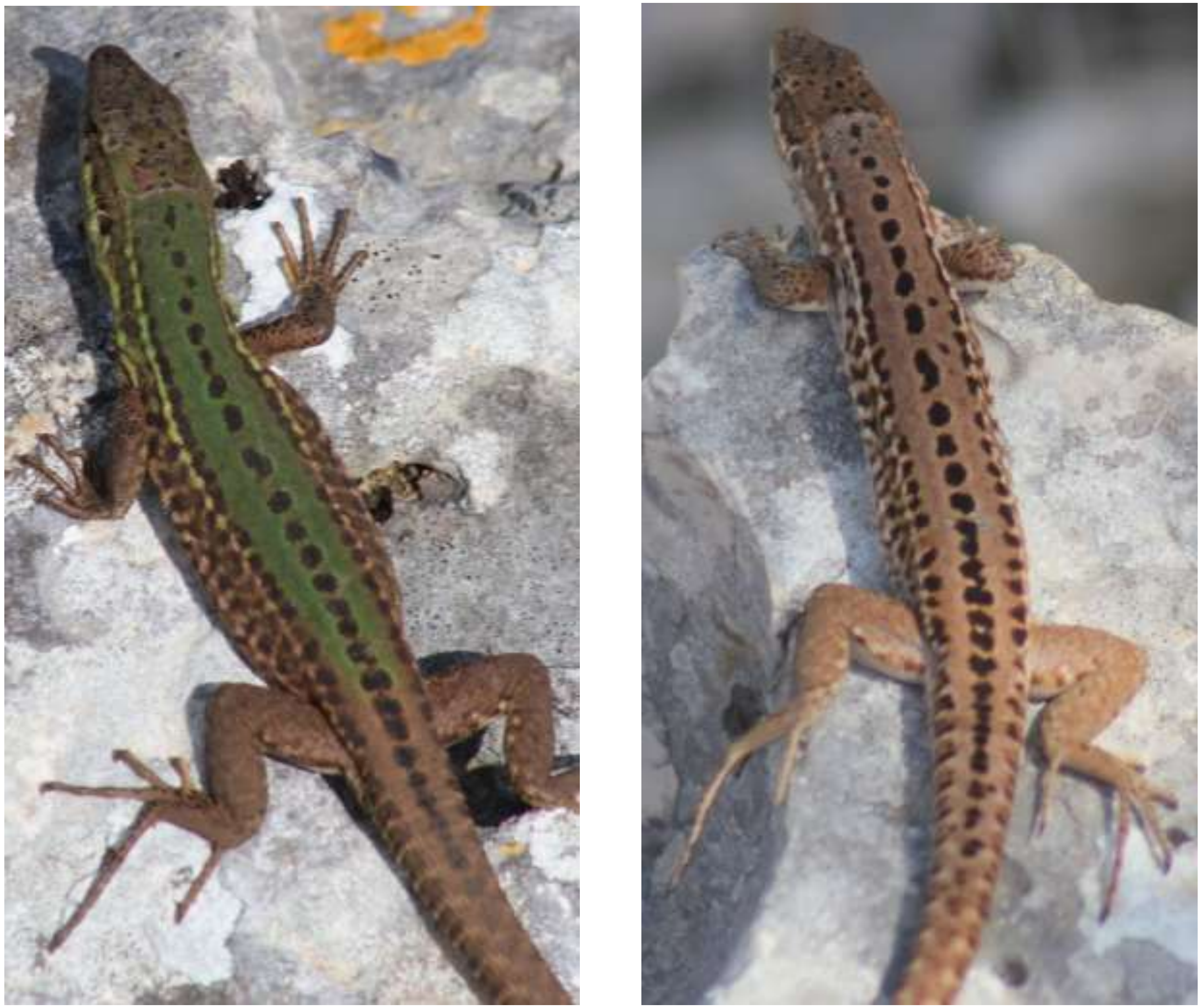

Figure s1.4 Female captured in March (left) and recaptured in August 2018 (right).

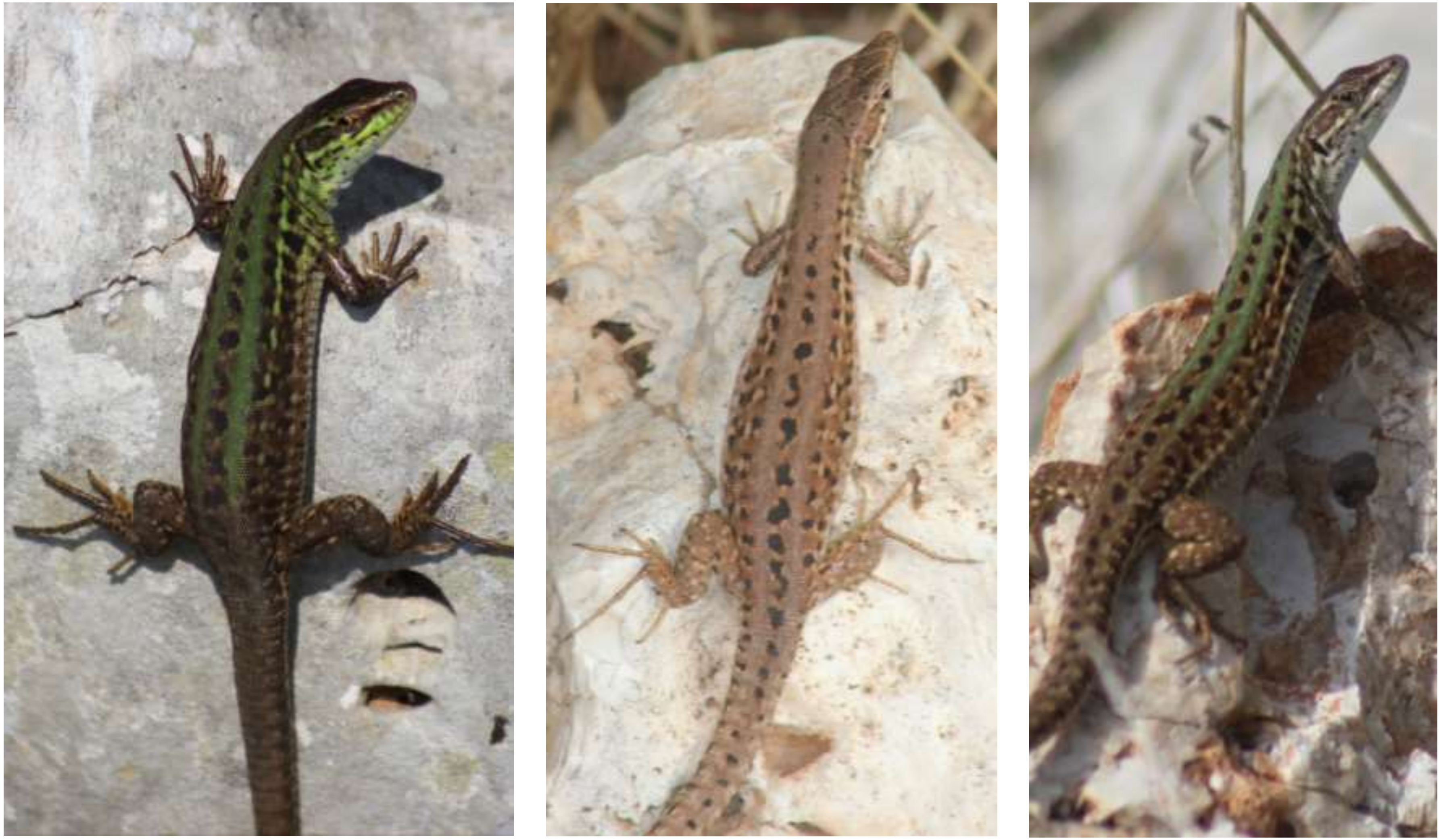

Figure s1.5 Female photographically captured in March (left) and recaptured in August (middle) and October 2018 (right).

Biology Open • Supplementary information

Table S1

Click here to Download Table S1

Biology Open • Supplementary information
